# Comparison of Geographic Information System and Subjective Assessments of Momentary Food Environments as Predictors of Food Intake: An Ecological Momentary Assessment Study

**DOI:** 10.2196/15948

**Published:** 2020-07-22

**Authors:** Katherine G Elliston, Benjamin Schüz, Tim Albion, Stuart G Ferguson

**Affiliations:** 1 College of Health and Medicine University of Tasmania Hobart Australia; 2 Institute of Public Health and Nursing Science University of Bremen Bremen Germany; 3 Leibniz Science Campus Digital Public Health Bremen Germany; 4 Menzies Institute for Medical Research University of Tasmania Hobart Australia

**Keywords:** ecological momentary assessment, mHealth, geographic information systems, food intake, mobile phone

## Abstract

**Background:**

It has been observed that eating is influenced by the presence and availability of food. Being aware of the presence of food in the environment may enable mobile health (mHealth) apps to use geofencing techniques to determine the most appropriate time to proactively deliver interventions. To date, however, studies on eating typically rely on self-reports of environmental contexts, which may not be accurate or feasible for issuing mHealth interventions.

**Objective:**

This study aimed to compare the subjective and geographic information system (GIS) assessments of the momentary food environment to explore the feasibility of using GIS data to predict eating behavior and inform geofenced interventions.

**Methods:**

In total, 72 participants recorded their food intake in real-time for 14 days using an ecological momentary assessment approach. Participants logged their food intake and responded to approximately 5 randomly timed assessments each day. During each assessment, the participants reported the number and type of food outlets nearby. Their electronic diaries simultaneously recorded their GPS coordinates. The GPS data were later overlaid with a GIS map of food outlets to produce an objective count of the number of food outlets within 50 m of the participant.

**Results:**

Correlations between self-reported and GIS counts of food outlets within 50 m were only of a small size (*r*=0.17; *P*<.001). Logistic regression analyses revealed that the GIS count significantly predicted eating similar to the self-reported counts (area under the curve for the receiver operating characteristic curve [AUC-ROC] self-report=0.53, SE 0.00 versus AUC-ROC 50 m GIS=0.53, SE 0.00; *P*=.41). However, there was a significant difference between the GIS-derived and self-reported counts of food outlets and the self-reported type of food outlets (AUC-ROC self-reported outlet type=0.56, SE 0.01; *P*<.001).

**Conclusions:**

The subjective food environment appears to predict eating better than objectively measured food environments via GIS. mHealth apps may need to consider the type of food outlets rather than the raw number of outlets in an individual’s environment.

## Introduction

### Background

Consistent with the notion of stimulus control, momentary environments are key correlates of a range of health-risk behaviors. For example, studies have shown associations between exposure to smoking-friendly environments and smoking [1], being in an abandoned space and illicit drug use [2] and being closer to fast-food outlets and an increase in discretionary food intake [3]. As such, being aware of what is in an individual’s momentary environment could provide a means for issuing just-in-time adaptive interventions [4]. For example, when entering environments known to trigger health risk behaviors, mobile health (mHealth) technology could generate interventions and support to individuals [5], thereby minimizing the risk of engaging in health-damaging behaviors [6].

However, for effective, just-in-time, and geofenced intervention designs, it is crucial to know the components of momentary environments that are most reliably related to risk behaviors. In particular, it is an open question whether the subjective perceptions (eg, the number of food outlets an individual perceives as close by) or the objective indicators of food environments (eg, a geographic information system [GIS]–based count of the number of food outlets in a given radius around an individual) are more reliably associated with health risk behaviors, such as high-calorie snacking.

Previous real-time studies have typically favored self-reported measures, requiring a user to manually input details surrounding their affect, activities, and environment [1,3,7]. For example, many studies ask participants to indicate their current environment from several prespecified locations (eg, work, home, restaurant or bar). Intensive self-report is desirable in the context of research studies, but such monitoring is burdensome and, hence, likely to be unfeasible for the long-term usage that is necessary to achieve a lasting behavioral change. Although self-reported data might generate richer data sets, for example, by allowing researchers to gather data on unobservable psychological processes and motivations, this needs to be balanced against the possibility of missing data through noncompliance with monitoring protocols. Another option is to passively monitor an individual’s environment using location, movement, or biometric sensors. In the case of location, for example, this could be achieved by combining GPS data from individual devices with GIS data, which could then be used to create targeted geofence-based mHealth interventions. Being passive, such monitoring is likely better suited for long-term monitoring than relying on self-reported information.

Passive monitoring, however, is not without its potential drawbacks. Of particular concern is that passively collected GPS data and self-reported data may capture differential aspects of the environment that might be relevant for behavior change. For example, although passive monitoring may be objectively accurate, individuals may not always be aware of—or influenced by—cues in their surrounding environment. It is possible that being actively aware of environmental cues is crucial to the initiation of health risk behaviors; therefore, passively monitoring locations may not be an appropriate way to target context-sensitive interventions. Indeed, some studies explicitly ask individuals to report on their behavioral triggers using a *cues to action* scale [8,9], thereby implying that the individuals are aware of the environmental cues that trigger their behavior. Previous environmental interventions have been shown to improve health behavior, such as food safety behaviors [10], suggesting that consciously perceived cues can trigger behaviors. However, other behaviors, such as eating, maybe prompted by the automatic processing of environmental cues, such as advertisements and brand logos [11,12]. This is consistent with stimulus control theory as it does not specifically require conscious awareness of cues. Therefore, in this study, we obtained both passive and active measures of the environment and compared the associations of both with food choices, a behavior shown to be influenced by environmental cues [3,13-15]. Comparing potentially different effects of passive and actively collected location information will allow us to examine the automatic and deliberate processing of cues that may prompt individuals to eat.

Although the role of environmental determinants on eating behavior has been previously examined [16-18], these studies typically conceptualize a static notion of the environment by relying on postcode information to calculate estimates of food outlets—which can be viewed as a proxy measure for food availability—in the neighborhood food environment corresponding to the residential address of a particular person. However, each day, people move between different neighborhoods and do not always shop in their residential areas [19,20]. Therefore, studies need to consider environmental food exposures using individuals’ daily travel patterns (their *activity space* [21]). On the other hand, studies that examine fluctuating environmental exposures have captured the food environment using self-reported measures [3,21]. However, with developments in technology, it is increasingly possible to use GIS data to provide an objective measure of the environments to which individuals are exposed to throughout the day.

### Objectives

As ecological momentary assessment (EMA) [22] allows for real-time assessment of an individual’s environment, it might be a particularly useful technique for examining environmental exposures to food intake. This study, therefore, used EMA to examine the GPS coordinates of individuals as they go about their daily lives. As previous studies have supported the role of environmental cues prompting eating, this study aimed to extend this work by investigating whether objectively collected information on momentary environmental exposures (through automatic GPS reports) predict food intake as effectively as subjectively reported environmental cues.

## Methods

### Overview

This study was a part of a larger project designed to examine the relationship among attentional bias, stimulus control, and obesity and to explore BMI-related differences among individuals’ eating behaviors [23]. It used EMA methods to explore the feasibility of using GIS data to predict snacking. The participants carried a study-issued smartphone for 2 weeks to self-report their food and drink intake in real-time and respond to randomly timed prompts throughout the day (see Measurement Instruments below, for assessment details). During assessments, participants self-reported on environmental exposures, including describing the number and type of food retail outlets nearby. In addition to these self-reported responses, the smartphone logged the participants’ GPS locations. The participants’ GPS locations were then overlaid on a GIS map of known food outlets. Thus, the study obtained both objective (GIS) and self-reported information about the participants' environment at each time point. A comparison of the environments logged in the food reports with random prompts allowed for the examination of environmental cues to eating.

### Participants

Seventy-nine participants were recruited for this study by looking at everyday food choices through social media advertising and a university staff newsletter in Tasmania. The eligibility criteria included being above 18 years of age, not currently dieting, and having no history of an eating disorder. BMI was stratified to obtain equal groups of participants in the healthy weight range (BMI ≥18.5-24.9) and the overweight and obese (BMI ≥25) range. Upon the completion of the study, the participants received an AUD $60 (US $39.3) shopping voucher as reimbursement for their time. Ethics approval was obtained from the Tasmanian Social Science Human Research Ethics Committee (reference number H0017015).

**Figure 1:**
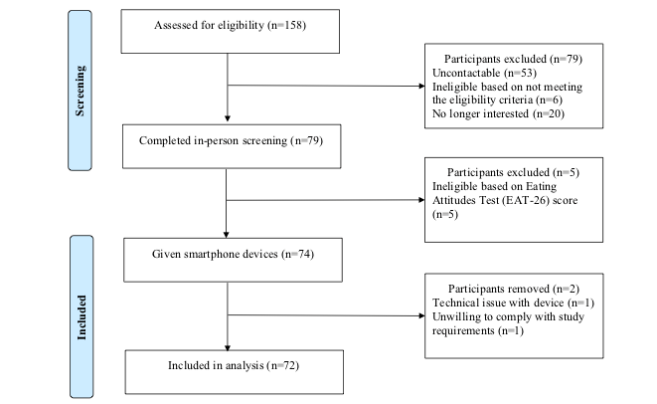
Participant flow diagram.

Five participants were excluded from the study because of screening scores exceeding 20 on the Eating Attitudes Test [24], indicating concerns regarding body weight, shape, and eating (Figure 1). In addition, 2 more participants were removed (1 participant was removed because of technical issues with his or her electronic device resulting in missing GPS stamps and 1 participant withdrew from the study). This left a total of 72 eligible participants, 71% were females (51/72; mean age 33.72 years, SD 12.08). BMI ranged from 18.59 to 40.22 (mean 26.67, SD 5.62). Most participants (62/72, 86%) were white. Over half, (43/72, 60%) of the participants had graduated from university, and 28% (20/72) participants had completed at least some university or were currently studying at a university. All participants lived in areas classified as urban [25].

### Procedure

Participants attended 3 study visits during the 14-day monitoring period. During the first visit, participants provided informed consent, were weighed, and their height was measured by study staff to calculate their BMI (kg/m^2^). Participants also completed a baseline survey assessing demographic information and their general dietary intake and received training on how to use the electronic diaries. Participants began recording their food intake; situational cues, such as their environment; and their affect levels immediately after this visit. During the participants’ second visit (around day 2-3 of monitoring), participants’ EMA data were uploaded and retraining was provided as necessary. During the third visit, after 14 days of monitoring, participants returned their study devices, were debriefed, and received reimbursement for their participation.

For the duration of the 2-week monitoring period, participants logged their food and drink intake and responded to the randomly timed prompts using a specially programmed smartphone. To reduce the participants’ burden, a random subsample (approximately 60%) of the food reports were followed by a set of questions assessing perceptions of the local food environment and contextual cues, such as the participants’ affect level and food cravings. In addition to the food reports, participants were issued a series of randomly timed prompts, occurring approximately 4 to 5 times per day. During the randomly timed prompts, participants received the same assessment questions as the food reports. The randomly timed prompts served as a comparison of situational and contextual details regarding eating versus noneating times. All the participants’ reports were time, date, and geographically stamped using a combination of GPS and mobile phone transmitter triangulation. Participants received an AUD $60 (US $39.3) shopping voucher upon completion of the study and the return of their EMA device, but they were not given additional payment for completing the randomly timed prompts.

### Measurement Instruments

*Food intake* was measured via participants’ self-reports. Participants reported—by tapping a button on the Android device—whether their food intake was a main meal or a snack.

Current environmental exposures were assessed via both subjective (self-reports) and objective reports (GPS stamps with subsequent GIS integration)—collected during participants’ randomly timed prompts and food reports. For the *self-reported food outlets*, participants were asked to report the number of food outlets nearby. Participants were presented with the question, “From where you are now, how many food outlets can you see?” Then, they were given a list of 6 types of food outlets: (1) fast-food and takeaway stores, (2) restaurants and cafes, (3) supermarket and corner store, (4) specialty food stores, (5) discount stores, and (6) other. Participants entered a number ranging from 0 to 5+ corresponding to each type of food outlet nearby (total possible range 0-30+). For model 2 in the analysis, the total number of self-reported food outlets within sight were summarized. For model 3 in the analysis, each self-reported outlet type was dichotomized (0=absent and 1=present), and all outlet types were simultaneously entered into the model.

For the *objective measure of food outlets*, the participants’ electronic devices automatically recorded their GPS coordinates every time they completed a report. The GPS location for each outlet and the participants’ locations were first split into latitude and longitude coordinates. The distance between the participants and the food outlets was calculated by overlaying their GPS coordinates with a combination of 3 local city council food outlet maps using Environmental Systems Research Institute’s ArcMap [26]. Local council food outlet maps were obtained, with each council providing the outlets’ names, addresses, and type of each food outlet. The councils classified food outlets as being a bakery, butcher shop, café, canteen, caterer, delicatessen, eatery, fish shop, food van, hotel, meat premises, restaurant, sports club, supermarket, takeaway, vessels selling food, or other. However, the classification of food outlets was not consistent across councils, which meant that the study was unable to separate food outlets into venue types. As a result, this study used an indicator of any food outlet near participants for analyses. Using council-reported latitude and longitude coordinates of local food businesses, food outlets within a 50-m radius of a participant’s GPS location were identified using the Buffer tool from the Analysis Tools Proximity toolbox [26]. The number of food outlets near a participant at the time of each report was then summarized and used in the analyses using the GIS measures.

### Analytical Procedure

To examine whether passively collected GPS reports correspond to the self-reported food environment measures, a repeated measures correlation between the GIS-derived counts and self-reported counts of nearby food outlets was calculated using the R package rmcorr [27]. Next, both GPS-derived food outlet and self-reported food outlet measures were used in participant-level logistic regression models to determine if the number of food outlets within the immediate environment discriminated between eating and noneating reports. Consistent with previous studies [28], the days on which random prompt compliance was below 50% were excluded from the analysis (total 145 days). Poor random prompt compliance may indicate instances of disengagement from the study protocol or systematic biases within the data and are, therefore, removed from further analysis.

While accounting for individual differences in eating, logistic analyses were conducted on each participant’s data to gauge the effect of the local food environment on food intake. First, a series of within-subject univariate logistic regression models using the area under the curve for the receiver operating characteristic curve (AUC-ROC) analyses were run. During the randomly timed assessments, each model examined if the odds of eating were higher when the density of food outlets was higher. For each model, food intake (yes or no) was the outcome variable and environmental measures (1) 50-m GIS food outlet count, (2) self-reported food outlet count, and (3) self-reported food outlet type were predictors. The study chose 50-m as it was a rough approximation of the line of sight typical for urban settings; thus, this radius was deemed as a reasonable approximation of the self-reported measure. AUC-ROC values can range from 0.5 (random guessing; no prediction) to 1.0 (perfect prediction), indicating the probability of identifying an eating event (versus a randomly timed prompt).

After generating an AUC-ROC for each participant for models 1 to 3 of the environment, the mean for each model was compared with 0.5 (ie, no predictability, at *P*<.05 threshold) using weighted *t* tests. This was used to determine the environmental measures that could accurately differentiate between eating and noneating (ie, randomly timed) assessments. Observations were weighted by the inverse of the SE of the AUC-ROC scores to allow more precise estimates to receive greater weight [29,30]. If the AUC-ROC score was significantly different from 0.5 at the *P*<.05 threshold, the model was able to accurately differentiate between eating instances and randomly timed prompts.

Next, 3 *t* tests were run to compare the food count models with each other and compare each count model with the self-reported outlet type. The *t* tests used each participant’s AUC-ROC score for the comparisons. Bonferroni adjustments were applied (at *P=*.02 level) to account for the inflation of type 1 errors with multiple comparisons. Finally, the correlations between GIS-derived measures and the self-reported measures were analyzed. This enabled the determination of the passively collected (ie, GIS-derived) environmental information was comparable with the environmental exposure information generated through self-reports. In addition, the counts between GIS-derived assessments of the food environment for both 50-m and 100-m surrounding an individual were compared, and the same basic outcomes were found. The results of the 50-m GIS count of only food outlets are presented below. All analyses were conducted in R version 3.3.1.

## Results

### Overview

Seventy-two participants completed between 3 and 21 days of EMA monitoring and were retained in the analysis: mean 14.74 (SD 2.58) monitoring days per person. In total, 1061 days of food intake and the immediate food environment were recorded. GIS measures recorded 2097 food outlets within a 50-m radius of the participants, and the participants self-reported a total of 1756 food outlets. Over the monitoring period, participants completed 3302 food reports, and 36.86% (1217/3302) of those were snacks. Participants reported between 2 and 10 food intakes (meals and snacks) per day (mean 4.42, SD 1.47). The snack intake ranged from 1 to 8 (mean 2.02, SD 1.24) per person per day. Participants received between 0 and 11 randomly timed assessments each day (mean 3.28, SD 1.73), and the compliance with the randomly timed assessments ranged from 35% to 100%. Overall, the compliance with the randomly timed assessments was excellent [31] (mean 78.75%, SD 14.75).

### Geographic Information System–Derived Measures of Food Outlets

**Figure 2:**
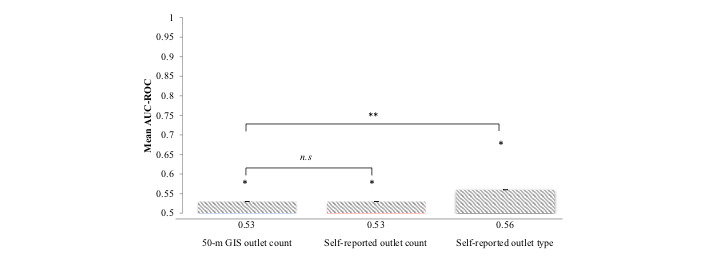
Mean area under the curve for the receiver operating characteristic curve (AUC-ROC) for each measure of the local food environment. The AUC-ROC value represents the probability of accurately differentiating between eating and noneating instances. The * symbol denotes t tests, where the mean AUC-ROC was significantly different from 0.50. The ** symbol denotes significant differences in AUC-ROC values (based on t-tests with alpha set to .02). n.s denotes models where the AUC-ROC values are not significantly different. Error bars indicate the SE for each model.

The GIS-derived AUC-ROC values ranged from 0.50 to 0.87 and yielded similar AUC-ROC values for the self-reported food outlet count (AUC-ROC for 50-m GIS food outlet count=0.53, SE 0.00; AUC-ROC for the self-reported food outlet count=0.53, SE 0.00; Figure 2). Weighted *t* tests showed that the GIS-derived model had AUC-ROC values significantly higher than 0.50 (the null value; *P*<.001), indicating that the presence of food outlets within a 50-m radius of an individual is significantly better than chance at discriminating between eating and noneating instances.

### Self-Reported Measures of Food Outlets

The AUC-ROC values for the self-reported count of the number of food outlets within sight ranged from 0.50 to 0.62 and had a similar AUC-ROC value for the 50-m GIS count (AUC-ROC for the self-report food outlet count=0.53, SE 0.00; AUC-ROC for the 50-m GIS food outlet count=0.53, SE 0.00; Figure 2), indicating that both measures of food outlets in the environment are significant predictors of food intake. Results from a paired sample *t* test showed no significant difference between the 50-m GIS count and the self-reported food outlet count on the participants’ AUC-ROC scores: t_71_=0.82, *P*=.41, and *d=*0.00.

The AUC-ROC for the self-reported type of food outlets in the environment ranged from 0.50 to 0.75. Model 3 showed that the self-reported type of food outlet was also a significant predictor of eating (AUC-ROC=0.56; Figure 2). A paired sample *t* test showed that there was a significant difference between the 50-m GIS model and the type of food outlets on the participants’ AUC-ROC scores: t_71_=−2.71, *P*<.001, and *d*=0.40. Similarly, there was a significant difference between the self-reported food outlet count and the type of food outlets on the participants’ AUC-ROC scores: t_71_=−5.16, *P*<.001, and *d*=0.48.

### Correlations Between Environmental Measures

A repeated measures correlation between the 50-m GIS food outlet count and the self-reported food outlet count was significant but weak (*r*=0.17; *P*<.001), indicating that assessments of the food environment derived through GIS are similar to the self-reported environmental measure. As the local councils had slightly different classifications of food outlets, the study was unable to compare the self-reported types of food outlets with a GIS-derived assessment.

## Discussion

### Principal Findings

This study used EMA methods to compare the assessments of momentary food environment using subjective and location stamp (GPS and GIS) data. The study found that the GIS-derived counts and self-reported counts of food outlets performed worse than the self-reported type of food outlets at predicting eating. These results suggest that subjective assessments of food outlet type are better predictors of momentary food intake and that the objective and subjective counts of food outlets may capture conceptually different aspects of the food environment compared with the subjective outlet type.

The finding that the type of food outlet nearby influences eating is consistent with findings from previous literature [32,33] and is likely to be evidence that food outlet density is a proxy measure for the availability of food. For example, living within one mile of a grocery store has been associated with increased fruit and vegetable intake [18] and having numerous supermarkets in one’s neighborhood is associated with lower BMI [34]. However, other types of food outlets are associated with increased unhealthy eating. For example, greater access to fast-food restaurants has been associated with a higher likelihood of fast-food purchasing [35] and a higher risk of overweight and obesity [16,34,36]. Overall, this suggests that the type of food outlet in the environment influences individuals’ diet and weight. Importantly, however, much of this previous research has relied on static assessments of individuals’ environments, that is, their residential addresses. This research, however, examined momentary environments, thereby accounting for the fluctuations in the environments to which individuals are exposed to throughout the day, each day.

The finding that subjectively reported food outlet counts and objectively reported food outlet counts are equally predictive of behavioral indicators (ie, eating) is novel. In the domain of physical activity, research has examined static environments and found inconsistencies between the availability and accessibility of parks to an individual and engagement in park-based physical activity [37,38]. Assessing park proximity and acceptability (ie, transport to parks, park paths or trails, and park cleanliness) differ based on whether the assessments are subjectively or objectively reported [38]. Given that the assessments of the environment are differentially associated with park-based physical activity based on the measurement type, it is possible that objectively and subjectively reported information may be tapping into conceptually different exposures. In terms of the momentary food environment as examined in this research, triggers to the craving and subsequent food intake may depend on the type of food outlet in an individual’s immediate environment; such information is not captured through counts of nearby food outlets. Certain food outlets (eg, fast-food restaurants) may be more likely to trigger cravings than other food outlets (eg, supermarkets) as the sights and smells from these outlets are associated with highly palatable food [39]. Therefore, subjectively reported food outlets—specifically, the type of food outlets nearby—may be better predictors of eating than count-based assessments of the food environment.

Despite finding a small correlation between the self-reported food outlet count and the 50-m GIS count, there is minimal difference between subjective and objective measures of the number of food outlets within the environment. Overall, the results of this study suggest that the type of food outlet nearby is a better predictor of eating (versus noneating) than the density or number of food outlets. The difficulty with this is that there is a lack of standardization with the classification of food outlets. For example, an outlet can be classified as a butcher shop in one council and as a meat premise in another. For this reason, the study was unable to calculate GIS-based assessments of food outlet types. Therefore, passively collected data with subjective assessments of various food outlet types on eating could not be compared. Importantly, neither the objective nor the subject measure can be considered a truly *accurate* measure of food outlet density; both measures involve a degree of measurement error. As such, although it can be concluded that the 2 measures are aligned, the differences between the measures as evidence for over- or under-reporting of the subjective values cannot be used.

If the information on the food outlet types were measured consistently across councils, mHealth apps may be able to passively monitor an individual’s location and proactively issue interventions before dietary lapses occur. This could be useful given this study’s finding that subjectively reported food outlet type is a better predictor of momentary food intake than either of the count-based measures. Alternatively, mHealth apps may be able to create personalized GIS maps of environmental triggers to eating by relying, at least initially, on subjective user input. Users could report their eating locations, and the corresponding GPS reports could be used to determine the locations where the users are most likely to consume unhealthy foods. When locations are repeatedly associated with unhealthy food intake, mHealth apps could then deliver just-in-time adaptive interventions to users.

The presence of restaurants, in particular, maybe a target for mHealth dietary apps using geofencing techniques. The energy content from meals consumed at restaurants has been found to contribute to most daily energy requirements [40]; thus, the presence of restaurants may be an appropriate target to reduce daily energy intake. Furthermore, some individuals may be particularly susceptible to eating unhealthy foods only when out [41]. Although this study did not examine the within-person differences in the healthiness or energy intake derived from food intake when out, it was able to examine how eating can be prompted by cues in the immediate environment. Future studies should examine person-specific traits that increase vulnerability to unhealthy eating when out.

Overall, the findings of this study suggest that an individual’s eating can be predicted based on his or her momentary environment. Although the self-reported type of food outlet nearby was the superior model in predicting eating, it only differentiated instances of eating versus noneating 56% of the time. It is possible that geofencing-based information may not be the best way to predict eating. However, research has demonstrated a relationship between the immediate food environment and individuals’ food intake; therefore, the examination of whether subjectively reported environmental information is comparable with GIS-derived data provides a starting point toward creating simple user-friendly mHealth dietary interventions.

Although using GIS data for mHealth dietary interventions passively collects data and is, therefore, less burdensome for users, it is particularly time-consuming to code, placing the burden instead on the app developers. However, once GIS data have been coded, the process of data collection becomes automated, whereas subjectively reported information will continue to require manual intervention from the user. In addition, GIS maps can be calculated once and rolled out across multiple studies and numerous sites. Such wide-scale use of location information is easier with automated GIS data than subjectively reported data. Nevertheless, the costs and benefits of each method must be balanced between users and app developers.

The finding that the overall predictive ability of the presence of food outlets on predicting eating was modest is consistent with the idiosyncratic nature of how cues come to be associated with behaviors. For example, eating could be highly related to a particular cue for one person, but different cues will be important for other people. On the basis of these findings, for relevant individuals, it may be beneficial to issue personalized dietary interventions when they enter environments where they are most at risk of overeating or unplanned eating. Indeed, similar geofenced interventions have been successfully trialed in the literature for smoking (eg, the Q-Sense app [42]). Q-Sense delivered support to users based on a 100-m geofence from a location where the user reported smoking on at least 4 occasions [42]. It appears that mHealth apps may need to rely (at least initially) on user input to create relevant geofenced risk areas and, subsequently, generate place-based interventions. Importantly, research to date demonstrates that environmental interventions are feasible, and users report no privacy concerns with location-based data monitoring [42].

### Strengths, Limitations, and Future Research

This study has several strengths. To the researchers' knowledge, this study is the first to integrate 2 ways of assessing the effect of an individual’s immediate food environment on his or her food choices. By using both objective measurements of the environment and subjective reports, we were able to compare how momentary environmental exposures influence real-time eating decisions. Such information provides a greater understanding of how individuals’ dietary choices may be influenced by momentary environmental cues.

The use of EMA to assess eating and food environment enabled the examination of real-time environmental exposures and how they influence eating decisions. Previous studies [20,43] have highlighted the need to use spatial data to examine environmental exposures and develop precise estimates of where individuals travel and purchase foods. The use of GIS data in this study allowed for a better understanding of how fluctuations in the momentary food environment shape an individual’s food choices. Furthermore, repeatedly assessing an individual’s environmental exposures allows for in-depth information on the environmental antecedents and consequences of overeating and dietary lapses.

The use of real-time reporting of food intake means that the participants in this study reported their current situation, activities, and environmental exposures and were, therefore, less prone to biases associated with recall [44]. Once behaviors are examined in real-time, an effective way of managing health-risk behaviors may be through issuing just-in-time adaptive interventions [6]. Just-in-time adaptive inventions may be able to utilize real-time cues, such as GPS-based information to identify individuals entering high-risk situations that require intervention and behavioral support (eg, the A-CHESS app [45]). The real-time aspect of this study is, therefore, the first step in identifying ways to conceptualize the environment to inform just-in-time adaptive interventions and mHealth apps.

Despite these strengths, there are some methodological limitations to this study. First, calculating GIS counts of food outlets from local council areas is difficult, and the GIS data are not sufficiently detailed to illustrate what types of food outlets exist. Furthermore, the local councils included in this study had different classification systems for recording food outlets, which meant that comparison among various council districts was feasible only by looking at the summary rather than the type of food outlets. Ideally, the best way to geocode food outlets would be to use a combination of council data, Google maps data, and by visiting neighborhoods of interest to identify the type of food outlets present. Despite this being the ideal way to assess food outlets within the local environment, it would be extremely time-consuming and perhaps impractical in large cities with numerous food outlets. Future studies should explore different ways to classify the food environment so that the best and simplest measures are identified.

Second, by relying on food outlet counts (either GIS-derived or self-reported), the understanding regarding exactly what aspects of food outlets influence food choice was limited. Furthermore, this study did not separately examine the effect of each type of food outlet. Food choice is likely to be shaped by factors that are independent of food outlets, such as individual taste preferences and social norms [46], as well as the availability and affordability of foods [47], none of which are captured by assessing the counts of nearby food outlets. Further investigation into the availability and other choice determinants associated with food selection are warranted to investigate the aspects of food outlets that influence food choice.

Third, by focusing on GIS counts of food outlets, this study was unable to determine the food outlets and food-related cues that the individuals could see. There may have been times when the food outlets were in close proximity to the participants but were hidden from view. For example, there may be food outlets between buildings or hidden within lanes or buildings. As individuals’ decisions relating to food choice are thought to be shaped by momentary exposures to food cues [48], in situations where individuals cannot see nearby food outlets, they are unlikely to be influenced by their presence. Future research should consider other environmental exposures, such as advertising and food smells, in addition to the presence of food outlets in prompting individuals’ food choices.

Fourth, as noted earlier, this study chose 50 m as the radius as it was a rough approximation of the line of sight typical for urban settings. What someone can see from their current position will vary from place to place; this would have introduced error into this measure. Further work is required to determine the *optimal* unit of measurement; furthermore, it may prove fruitful to vary this measure from location to location based on the characteristics of the site.

Finally, the food outlet data from the local councils may not have been up-to-date. It is possible that there may have been a discrepancy between the GIS-derived food outlet count and the food outlets that were around and open during the time the study was conducted. Encapsulating the most recent and accurate information on the presence of food outlets is necessary to examine the association between the presence of food outlets and eating. Furthermore, this study did not consider the availability of food within each outlet. Factors like product availability and opening hours are likely to influence individuals’ food options and, subsequently, their eating decisions. mHealth apps that require user input on environmental eating triggers will likely circumvent this issue. At present, mHealth interventions are unable to achieve targeted place–based information with passively collected data.

### Conclusions

Examining the food outlets within one’s environment is an important step in understanding how the built environment influences eating. This study found that although passively knowing an individual’s environment can predict eating, knowing what type of food outlets are nearby is the best way for mHealth apps to create geofenced dietary interventions. Future advances in technology may enable passive calculation of the type of food outlets within a given geographical region. Such information would be integral to the success of geofenced interventions in mHealth dietary apps. In the meantime, mHealth apps will likely need to continue relying on users’ self-reported information about their food environment to generate tailored geofenced dietary interventions.

## Abbreviations

AUC-ROC: area under the curve for the receiver operating characteristic curve
EMA: ecological momentary assessment
GIS: geographic information system
mHealth: mobile health
